# Design and implementation of a microlearning-based OSCE preparatory curriculum in obstetrics: a pilot study

**DOI:** 10.1007/s00404-025-08157-6

**Published:** 2025-08-25

**Authors:** N. Michlmayr, J. Mitofsky, N. Haverkamp, A. Wittek, R. Plöger, A. Walter, B. Strizek, F. Recker

**Affiliations:** 1 Faculty of Medicine Bonn, Rhenish-Friedrich-Wilhelms-University Bonn, Venusberg Campus 1, 53127 Bonn, Germany; 2 Department of Obstetrics and Prenatal Medicine, University Hospital Bonn, Venusberg Campus 1, 53127 Bonn, Germany; 3 Office of the Dean of Studies, Faculty of Medicine Bonn, Rhenish-Friedrich-Wilhelms-University Bonn, Venusberg Campus 1, 53127 Bonn, Germany

**Keywords:** Medical Education, Microlearning, Obstetrics, OSCE preparatory curriculum

## Abstract

**Background:**

Bedside teaching in obstetrics is increasingly challenged by clinical workload, leading to inconsistent OSCE preparation for medical students. To address this, we developed and implemented a microlearning-based preparatory curriculum using the MiLeMed app.

**Methods:**

A microlearning-based OSCE preparatory curriculum was developed using Kern’s six-step model. Goals and objectives were defined via a Delphi process involving educators and students. A dedicated app with a 20-day study plan was created, piloted by 13 students, and evaluated using an online questionnaire.

**Results:**

The Delphi process supported the use of microlearning with integrated quizzes and visuals. Pilot participants reported the app as helpful for understanding and exam preparation, and emphasized its usability and inclusive design in open-text feedback.

**Discussion:**

The pilot study suggests that a microlearning app is a feasible and well-received tool for OSCE preparation. However, limitations such as small sample size, self-selection bias, and technological disparities must be considered. Future studies, including a planned randomized controlled trial, are needed to evaluate effectiveness and generalizability.

## Background

High workloads, fast-paced clinical environments, and low gratification are common challenges for many physicians in Germany [1]. In addition to their clinical duties, physicians are often expected to teach medical students during bedside instruction, despite limited time and resources. At the University of Bonn, medical students in obstetrics participate in bedside teaching that culminates in an Objective Structured Clinical Examination (OSCE), a format introduced in 1975 in the United Kingdom. During the OSCE, students rotate through stations and are assessed on specific clinical skills using standardized criteria. [2].

To address the dual challenges of limited teaching capacity and the need for standardized OSCE preparation, we developed a mobile microlearning curriculum delivered via the MiLeMed app. The app was developed at the University of Bonn by our team and designed to provide an accessible, time-efficient, and scalable educational solution that supports students without adding significant workload for instructors.

Microlearning enables flexible, asynchronous learning through short, focused modules accessed via smartphones [3]. While its adoption is growing in other fields, its integration into medical education—especially in obstetrics—remains limited [4, 5]. To our knowledge, no prior study has implemented a mobile microlearning curriculum to support OSCE preparation in obstetrics.

Against this background, we aim to explore whether an app-based microlearning preparatory course can be effectively implemented to enhance students’ preparation and perceived readiness for the Obstetrics OSCE. Although the present work primarily focuses on the design, implementation, and initial reception of the curriculum, future studies will address this question through structured evaluations of educational outcomes.

This study presents the design, implementation, and initial evaluation of a novel microlearning curriculum, developed using Kern’s six-step approach [6]. The app-based program was launched as a pilot at the University of Bonn and includes short lectures, images, interactive quizzes, and a structured 20-day study plan aligned with national educational standards.

## Methods

The development of the preparatory curriculum followed Kern’s six-step approach to curriculum development, a widely used framework in medical education. This model includes the following sequential steps: problem identification and general needs assessment, targeted needs assessment, formulation of goals and objectives, selection of educational strategies, implementation, and evaluation and feedback. [6] Each step was systematically addressed to ensure a structured and evidence-informed development process.

**Fig. 1:**
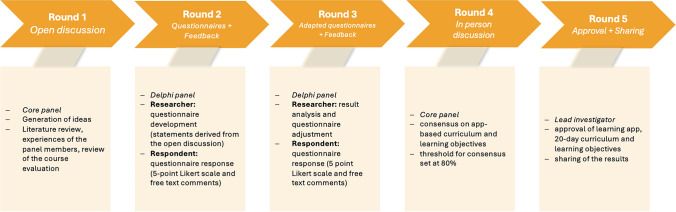
Delphi process

To guide the development process, we first reviewed results from previous internal course evaluations and conducted informal feedback discussions with medical students. These initial insights highlighted recurring challenges in OSCE preparation and served as the basis for a structured Delphi process to further define educational goals and objectives for a preparatory tool. (see Fig. 1)

A panel consisting of ten individuals—experts in medical education and obstetrics as well as student representatives—was convened at the University of Bonn. The aim of this process was to establish a consensus-based foundation for the development of a new preparatory resource for the Obstetrics OSCE. The Delphi procedure included four iterative rounds. Data collection was carried out through multilevel, self-administered questionnaires and free-text responses. In the first round, the panel unanimously agreed on the need to develop a digital resource to complement OSCE preparation. Based on the input collected, a set of structured statements was generated and subsequently rated by all panel members using a 5-point Likert scale. Additional comments allowed for qualitative refinement of the items. The consensus threshold was predefined at 80%. By the end of the fourth round, the panel reached agreement on key educational goals, content priorities, and implementation strategies. A final round of feedback and discussion served to confirm and finalize the decisions.

**Fig. 2:**
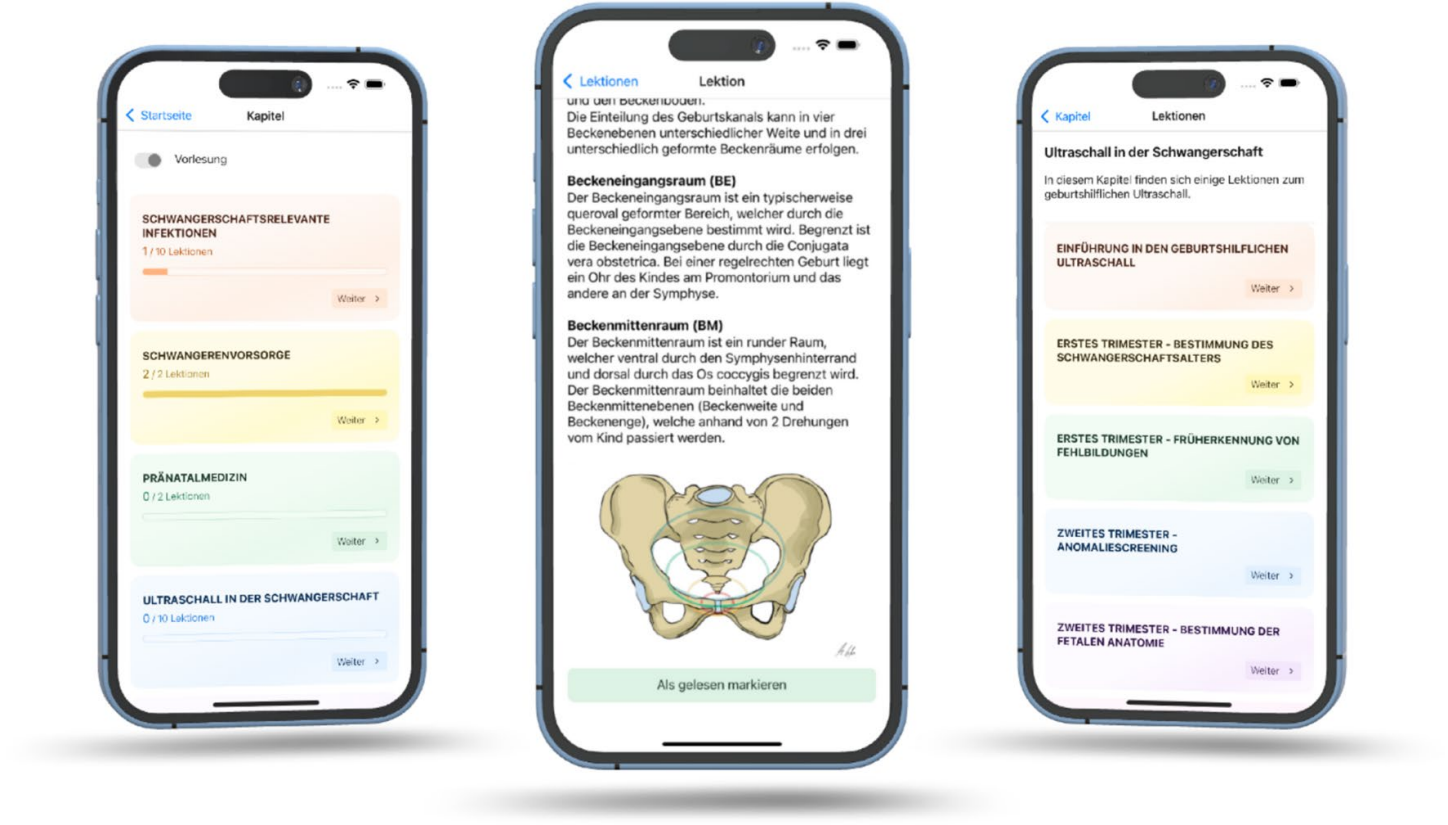
Visual Design and Learning Features of the MiLeMed Application

The app was developed in close collaboration with a software developer affiliated with the University of Bonn. The project team was responsible for defining the functional requirements, structuring the user interface, and populating the app with educational content. In addition to developing the educational content, we also created original visual materials, with particular attention to inclusivity in terms of representation, including ethnic diversity (see Fig. 2).

A beta version of the app was tested by a pilot cohort of 13 students during the obstetrics lecture series. These students did not use the app for OSCE preparation but rather as a supplementary tool for the ongoing obstetrics curriculum. No structured study schedule was provided; instead, students were encouraged to explore the app autonomously for pre- and post-lecture reinforcement. Following the semester, participants were invited to complete an anonymous online evaluation via the EvaSys platform. The questionnaire included several items measured on a 5-point Likert scale and one open-ended question. The primary focus of the evaluation was to assess user engagement and usage patterns. The quantitative data were analyzed using descriptive statistics, including median, mean, and standard deviation. In addition, free-text comments were thematically evaluated. Data analysis was conducted using the EvaSys system (evasys GmbH, V10.0).

## Results

### Step 1: Problem identification and general needs assessment

Clinical teaching in obstetrics is shaped by a range of structural factors, including the involvement of multiple instructors and the concurrent clinical workload of teaching staff. These conditions contribute to variability in both the content and consistency of instruction. Internal course evaluations and student feedback from previous semesters have indicated that preparation for the OSCE is often inconsistent, particularly with regard to theoretical knowledge. Informal consultations with teaching staff supported these observations. Moreover, students tend to enter the course with differing levels of prior knowledge and clinical experience, resulting in further heterogeneity in exam readiness. In light of these findings, there appears to be a need for a resource to support uniform theoretical preparation and to facilitate equitable exam readiness for all students.

### Step 2: Targeted needs assessment

As previously established, there is a need for a tool that supports effective theoretical preparation for the OSCE. The next step is to define the specific targeted needs that such a resource should address. To ensure educational relevance and practical utility, it should align with current medical education standards as defined by the German Medical Licensing Regulations (ÄAppO) [7] and the National Competence-Based Catalogue of Learning Objectives for Medicine (NKLM) [8]. Moreover, the tool should be easily accessible, adapted to the local curriculum at the University of Bonn, and structured to provide foundational theoretical knowledge related to the key clinical conditions and examination techniques covered in the OSCE [2].

### Step 3: Goals and objectives

As outlined in the methods section, a structured Delphi process was conducted to define the educational goals and objectives of the planned OSCE preparatory tool. The overarching goal identified by the expert panel was to provide students with a uniform theoretical foundation for their preparation, thereby addressing the heterogeneity in prior knowledge and teaching formats.

One specific objective was to integrate a digital tool that reflects the realities of modern medical education. The tool was expected to be easily accessible, user-friendly, and to minimize additional workload for students. After discussing various implementation options—including existing e-learning platforms—the panel reached consensus on developing a dedicated microlearning application. This approach was selected due to its flexibility, brevity of content delivery, and potential for high student engagement. It was also seen as an opportunity to introduce an innovative, app-based alternative to traditional learning formats.

Another key objective was to ensure alignment with current educational standards at both the national and institutional levels. Accordingly, the app content and structure were based on the *National Competence-Based Catalogue of Learning Objectives for Medicine* (NKLM) [8] and the *German Medical Licensing Regulations* (ÄAppO) [7]. In addition, the existing obstetrics curriculum at the University of Bonn was used as a content framework to ensure consistency with local teaching objectives.

To provide students with a structured framework for learning, the panel agreed on implementing a 20-day study plan embedded within the app. This format was chosen to promote self-directed yet guided learning and to support time management in the lead-up to the OSCE. The decision was further supported by the fact that such study plans are commonly used by medical students in Germany when preparing for state examinations, thus offering a familiar structure that enhances usability and acceptance.

### Step 4: Educational strategies

Within the framework of the Delphi process, the expert panel also defined the educational strategies to be implemented in the preparatory tool. The selected approach was based on evidence-informed principles designed to enhance student engagement, knowledge retention, and self-directed learning.

Microlearning was chosen as the core instructional strategy. This method is based on the principle of *chunking*, which breaks down complex or extensive information into smaller, manageable learning units. [9] In addition to reducing cognitive load and improving retention, the microlearning format was specifically designed to fit into the daily routines of students. By keeping individual lessons short, the app allows for learning in brief intervals—for example, while waiting at a bus stop—thereby promoting a high degree of temporal and situational flexibility.

To further support deep learning, elements of Mayer’s *Cognitive Theory of Multimedia Learning* were integrated into the instructional design. Text-based content was consistently combined with relevant visual elements to leverage the dual-channel processing of information, which is known to enhance comprehension and long-term memory formation [10].

In addition, each lesson concluded with a brief quiz to promote active engagement with the material. This shift from passive reading to active recall is a well-established strategy for reinforcing learning and improving knowledge retention. [11] The inclusion of formative assessments also allowed learners to self-monitor their progress [12].

Finally, gamification elements were incorporated into the app design to increase motivation and user interaction. These were implemented through both the visual and functional structure of the app, including the use of quizzes as interactive components. The combination of microlearning, multimedia integration, and low-threshold formative testing formed a cohesive and learner-centered educational strategy tailored to the needs of medical students preparing for the OSCE.

## Step 5: Implementation

The content of the app was derived from and closely aligned with the existing obstetrics curriculum at the University of Bonn. Core topics and competencies were identified and transformed into concise microlectures, each addressing a specific clinical condition or examination technique relevant to the OSCE. These microlectures were supplemented with both multiple-choice questions and open-ended reflection prompts to promote active engagement and self-assessment.

A structured 20-day study plan was developed to guide students through the content in a sequential and time-efficient manner. Both the app and the study plan were made available to students in preparation for the OSCE. To support the roll-out, an optional in-person information session was held, during which the app functionality, structure of the study plan, and intended use were explained. This session also provided an opportunity for students to ask questions and clarify expectations.

In parallel, teaching staff involved in OSCE preparation and bedside instruction were informed and introduced to the tool to ensure transparency and curricular alignment. This supported the integration of the app into the broader teaching context and facilitated its acceptance among instructors.

## Step 6: Evaluation and feedback

**Fig. 3:**
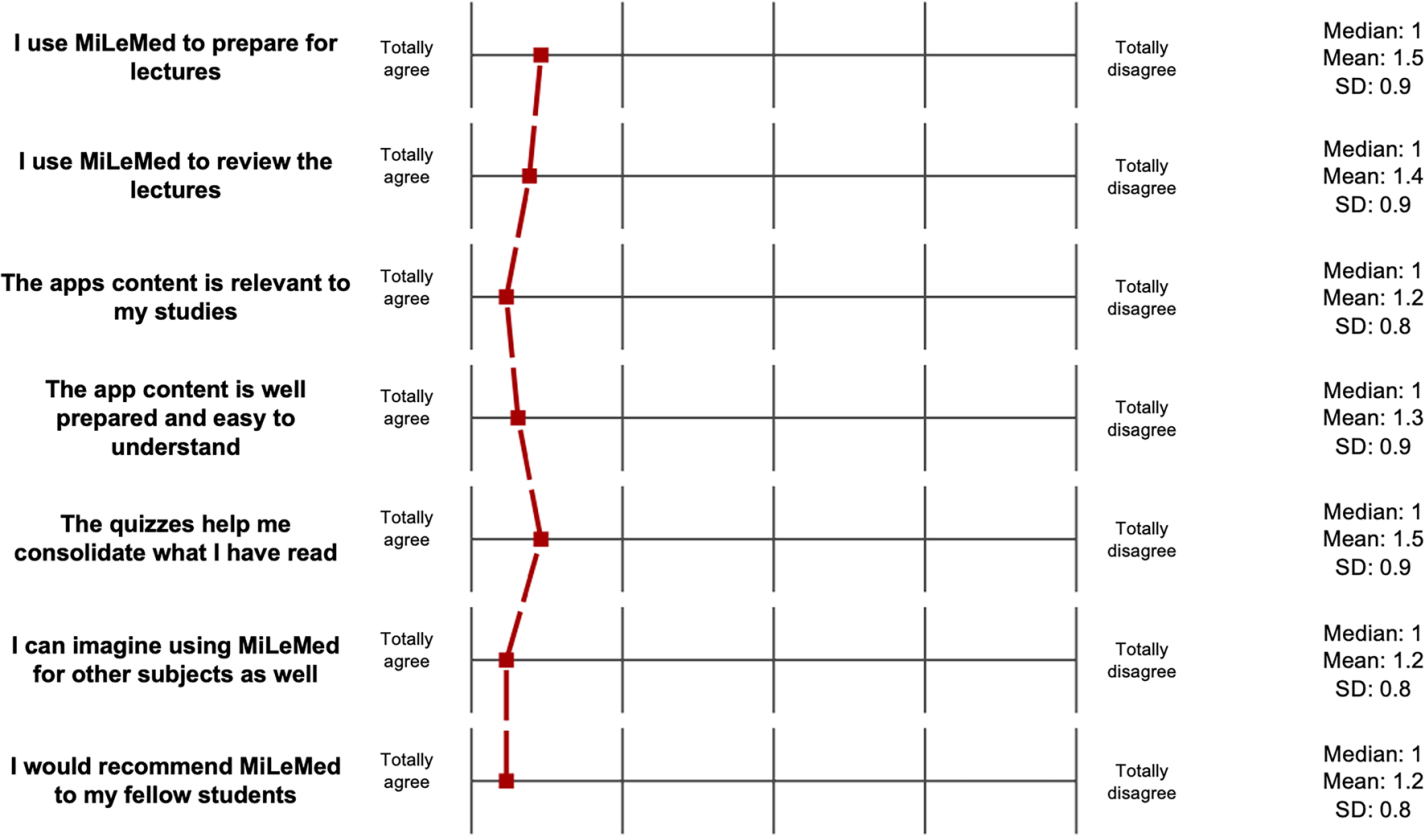
Feedback analysation (Descriptive parameters of the likert scale (1 = Totally agree, 2 = Agree, 3 = No orientation, 4 = Disagree, 5 = Totally disagree) *SD* Standard deviation

The feedback obtained from the pilot cohort following the beta testing phase of the app was predominantly positive. Students reported that the app served as a valuable complement to the obstetrics lecture series and supported their preparation for examinations. They indicated that the content was understandable and clearly relevant to the course objectives (see Fig. 3).

In the open-ended responses, several students expressed a desire for similar digital learning tools to be made available for other subjects within the curriculum. Furthermore, two students specifically highlighted the added value of the accompanying visual materials, noting that the illustrations were both pedagogically helpful and positively received in terms of their ethnic inclusivity.

## Discussion

The OSCE preparatory course in microlearning format, delivered via the MiLeMed app, was developed to enhance exam preparation and standardize students’ access to high-quality educational materials in obstetrics. This approach aims to supplement clinical teaching in an increasingly time-constrained healthcare environment.

Microlearning was deliberately chosen as the core instructional format due to its unique ability to deliver structured theoretical knowledge in a highly accessible and flexible manner. As smartphones are widely available among students, the app-based delivery of content ensures ease of access and allows integration into everyday routines. This format offers temporal flexibility by enabling short learning sessions throughout the day—whether between clinical duties or during commuting. Furthermore, microlearning reflects current technological trends in medical education and supports asynchronous, individualized learning. Beyond these pedagogical advantages, we also sought to explore a novel, innovative format that had not yet been applied to OSCE preparation in obstetrics in this form.

Alternative implementation strategies were considered during the planning phase. One such option was to compile a study plan utilizing existing learning platforms already in use at German medical faculties. However, this approach was ultimately dismissed, as it lacked institutional specificity and did not provide the same degree of temporal flexibility. Moreover, existing platforms were not originally designed to support structured OSCE preparation in the context of our local curriculum.

Despite the promising design and positive initial feedback, several limitations of our approach must be acknowledged. First, potential technological barriers could affect student access and engagement. Not all students possess high-performance smartphones or have reliable internet connectivity, which could introduce disparities in usage. Second, the quality and didactic effectiveness of the app content depend heavily on the materials generated by local instructors. Without consistent instructional standards, the usability and impact of the app could vary considerably. Third, student fatigue is an important consideration; students are already navigating multiple digital platforms, and the addition of another tool may contribute to cognitive overload or disengagement. Furthermore, while microlearning aims to reduce cognitive burden by breaking down content, the cumulative cognitive load of frequent interactions over the course of a semester remains insufficiently studied and could differ from traditional learning methods.

In addition, the feedback obtained from the pilot phase is limited in scope. The test cohort was small and not representative of the wider student population. As participation was voluntary and the data self-reported, results may have been influenced by social desirability bias or selection effects.

Future research is needed to evaluate the educational effectiveness of the microlearning curriculum. A randomized controlled trial is currently being planned, in which students will be allocated to two cohorts: one using the app as part of their OSCE preparation, and the other following traditional preparation methods. At the end of the semester, both groups will complete a structured questionnaire to assess subjective learning behaviors, satisfaction, and perceived preparedness. This will allow for a more systematic comparison and help determine the app’s added value in supporting OSCE readiness. In future studies, we also intend to include objective measures such as OSCE performance scores and app usage statistics to complement self-reported data and gain a more comprehensive understanding of learning outcomes. We are aware, however, that this type of self-assessment inherently carries limitations and must be interpreted with caution. Subjective measures are prone to bias, and their validity in capturing actual learning outcomes should be critically examined in future analyses.

## Data Availability

No datasets were generated or analysed during the current study.
